# Methods for detecting Zika virus in feces: A case study in captive squirrel monkeys (*Saimiri boliviensis boliviensis*)

**DOI:** 10.1371/journal.pone.0209391

**Published:** 2018-12-20

**Authors:** Krista M. Milich, Benjamin J. Koestler, Joe H. Simmons, Pramod N. Nehete, Anthony Di Fiore, Lawrence E. Williams, Jaquelin P. Dudley, John Vanchiere, Shelley M. Payne

**Affiliations:** 1 Department of Anthropology, Washington University in St. Louis, St. Louis, Missouri, United States of America; 2 Department of Anthropology, University of Texas at Austin, Austin, Texas, United States of America; 3 Department of Molecular Biosciences, Institute for Cellular and Molecular Biology and LaMontagne Center for Infectious Disease, University of Texas at Austin, Austin, Texas, United States of America; 4 University of Texas MD Anderson Cancer Research Center, Bastrop, Texas, United States of America; 5 Department of Pediatrics, Louisiana State University Health Science Center at Shreveport, Shreveport, Louisiana, United States of America; VA-MD College of Veterinary Medicine, UNITED STATES

## Abstract

A strain of Zika virus (ZIKV) of Asian origin associated with birth defects and neurological disorders has emerged and spread through the Americas. ZIKV was first isolated in the blood of nonhuman primates in Africa and has been detected in the blood, saliva, and urine of a few catarrhine species in both Africa and Asia, suggesting that nonhuman primates may serve as both a source and a reservoir of the virus. The recent introduction of ZIKV to human populations in the Americas presents the potential for the virus to spread into nonhuman primate reservoirs. Thus, it is critical to develop efficient and noninvasive detection methods to monitor the spread of the virus in wild nonhuman primate populations. Here, we describe a method for ZIKV detection in noninvasively collected fecal samples of a Neotropical primate. Fecal samples were collected from two captive squirrel monkeys (*Saimiri boliviensis boliviensis*) that were experimentally infected with ZIKV (Strain Mexico_1_44) and an additional two uninfected squirrel monkeys. Nucleic acids were extracted from these samples, and RT-qPCR was used to assay for the presence of ZIKV using primers flanking a 101 bp region of the NS5 gene. In both ZIKV-inoculated animals, ZIKV was detected 5–11 days post-infection, but was not detected in the uninfected animals. We compare the fecal results to ZIKV detection in serum, saliva, and urine samples from the same individuals. Our results indicate that fecal detection is a cost-effective, noninvasive method for monitoring wild populations of Neotropical primates as possible ZIKV reservoirs.

## Introduction

The majority of emerging infectious diseases (EIDs) are zoonotic in origin, with transmission occurring between humans and wildlife [1,2]. Understanding nonhuman reservoirs of viruses and zoonotic transmission routes of pathogens is critical to controlling outbreaks of EIDs. One such EID is Zika virus (ZIKV), a single-stranded positive-sense RNA virus of the family *Flaviviridae*, which spread throughout South America, Central America, and Mexico in 2014–2017 [3,4]. ZIKV infection in humans presents as a mild dengue-like disease, but is associated with congenital microcephaly, Guillain–Barré syndrome, and other neurological disorders [5,6]. Little is known about the nonhuman reservoirs of ZIKV; however, the virus was first discovered in a nonhuman primate in Africa, and several species of Old World primates are known carriers [7–12]. The recent introduction of ZIKV to the Americas presents the possibility for anthroponotic transmission of the virus to Neotropical primates, which were previously not known to carry the disease. These primates could then maintain the virus and serve as a reservoir for human infection.

Neotropical primates have been identified as reservoirs of other flaviviruses that threaten human health, including yellow fever virus (YFV) and dengue (DENV) [13–15]; notably, these viruses can also be fatal in certain species of Neotropical primates [16,17]. Several studies of captive animals have shown that Neotropical primates are susceptible to ZIKV and present a variety of symptoms associated with the disease [18,19]. Preliminary evidence indicates that ZIKV has spread from humans to free-living and captive capuchins in Brazil [20]; however, this study relied on detection of ZIKV neutralizing antibodies, which are known to be cross-reactive with other endemic flaviviruses, particularly dengue [21,22]. Additionally, a recent study presents evidence that ZIKV is now endemic in *Callithrix* and *Sapajus* species in Brazil [23]. Therefore, it is imperative to develop an effective surveillance system to identify and monitor reservoirs of ZIKV in wild nonhuman primate populations [11,24].

ZIKV monitoring in wild nonhuman primates is limited by the lack of an efficient, noninvasive detection method. ZIKV was first isolated in the blood of a rhesus macaque in Uganda and has since been detected in the blood, saliva, and urine of a few Old World primates in Africa and Asia [7–12]. Methods for detecting ZIKV in blood, saliva, and urine have recently been validated for Neotropical primates [18,19], but collecting these types of samples from wild primates is challenging. Blood and saliva can only be collected if an animal is captured or darted. While urine can be collected noninvasively, collection is inefficient and can only be attempted under specific conditions that are often difficult to meet in the field. By contrast, noninvasively collected fecal samples of wild primates have been successfully used in a wide range of behavioral and physiological research, including studies of genetic relatedness, disease ecology, and socioendocrinology. The relative ease of collection compared to other methods makes the development of a fecal detection method for ZIKV a high priority. Currently, only one other study has reported successful ZIKV detection in feces, which focused on an African lineage ZIKV strain distinct from that now circulating in the Americas [18].

To establish a non-invasive method for identifying and monitoring ZIKV in wild primate populations, we tested a semi-quantitative fecal detection method using reverse transcription quantitative PCR (RT-qPCR). Here, we describe a method for detecting a Mexican lineage ZIKV strain in feces of captive squirrel monkeys. These findings represent a significant advancement in our ability to monitor ZIKV in wild reservoirs with implications for monitoring other EIDs.

## Methods

### Ethics statement

This study was performed in strict accordance with the recommendations described in the Guide for the Care and Use of Laboratory Animals and in accordance with the Office of Laboratory Animal Welfare and the United States Department of Agriculture. All animal work was approved in advance by The University of Texas MD Anderson Cancer Center’s (MDACC) Institutional Animal Care and Use Committee in Houston, TX (Protocol #0001528-RN00), and all studies were carried out at the Michale E. Keeling Center for Comparative Medicine and Research in Bastrop, TX (Keeling Center), which is accredited by the Association for Assessment and Accreditation of Laboratory Animal Care.

All procedures were performed by trained personnel under the supervision of veterinary staff. Fetal ultrasound examinations used manual restraint, and all other procedures were carried out under ketamine anesthesia. Every effort was made to ameliorate the welfare and to minimize animal suffering in accordance with the “Weatherall report for the use of nonhuman primates” recommendations. Animal health and welfare was monitored twice daily, and all animals were housed under controlled conditions of humidity, temperature, and light (12-hour light/12-hour dark cycles) in an animal biosafety level 2-qualified research room at the Comparative Medicine Research Building. All animals received three types of enrichment; social, food, and housing. All animals were housed in pairs to provide social partners. Animals were fed commercial monkey chow twice daily and water was available ad libitum. Daily food enrichment included various in-season fruits and vegetables, as well as frozen and dry forage provided in destructible enrichment devices. All animals were also provided multiple layers of perching and different travel paths through their housing as a form of enrichment. Euthanasia was performed under an anesthetic plane with ketamine/xylazine, using either sodium pentobarbital (100 mg/kg) or Beuthanasia Solution or equivalent at 1 ml/5 kg intravenously. All nonhuman primate euthanasia procedures were performed in accordance with the AVMA Guidelines for the Euthanasia of Animals (2013 Edition).

### Subjects

The subjects for this study were captive squirrel monkeys (*Saimiri boliviensis boliviensis*) being socially housed in pair groups at the Keeling Center. To reduce the number of animals being infected with ZIKV, we collected samples from animals that were inoculated with the virus for a parallel study on ZIKV and pregnancy. Two monkeys (one pair group, designated animal #5165 and animal #5574) were experimentally infected with ZIKV as described in Vanchiere et al. [19], and fecal samples were also collected from a second uninfected pair group that served as negative controls. Two pregnant females were each inoculated with a total of 7 x 10^5^ genome equivalents of ZIKV strain Mexico_1_44 divided into 10 subcutaneous injections of 100 μl per animal. The estimated gestation age at the time of inoculation was 31 days. Blood, saliva, urine, and fresh fecal samples (approximately 2 grams) were collected from the females every other day after inoculation for approximately two weeks and then again on day 29 and day 59. Fecal samples were collected from the uninfected pair approximately every other day for two weeks. Samples were stored frozen after collection until RNA extraction and analysis.

After inoculation, the two individuals remained well with no signs or symptoms of infection. At 26 days post infection (dpi), however, animal #5574 had intrauterine fetal demise, based on failure to detect heartbeat activity or fetal movement by high-resolution ultrasound. A C-section was performed to recover amniotic fluid, fetal tissues, and placenta. The amniotic fluid was clear. The fetal tissues were very fragile, but the placenta appeared healthy. The estimated post-mortem interval was 48–72 hours. Plaque assays were performed as previously described [19] and are included as an independent confirmation of ZIKV infection in the study subjects. Briefly, approximately 1 cm^2^ tissue was collected in 2 ml PBS and homogenized. The homogenate was filtered, and 100 μl supernatant was added to a monolayer of Vero cells and incubated for 72 hours.

### Sample RNA extraction and analysis

Viral RNA from blood was extracted and detected using RT-qPCR methods at the University of Texas MD Anderson Cancer Research Center according to Vanchiere et al. [19]. Viral RNA from urine, saliva, and feces were independently processed and analyzed at the University of Texas at Austin as follows. Viral RNA from saliva and urine was extracted using QIAamp Viral RNA Mini Kits (Qiagen) as per the manufacturer’s instructions. For RNA extraction from feces, the entire fecal sample was first suspended in 5 ml of 0.89% NaCl and vortexed for 1 minute. This slurry was then centrifuged at 4,000 x g in a swinging bucket rotor for 10 minutes, and the supernatant was filtered through a 0.22 μm filter. The filtrate was transferred to a 100k Microsep advanced centrifugal filter (Pall), and then subjected to centrifugation for 25 minutes at 4,000 x g in a swinging bucket rotor to further concentrate the sample to approximately 300 μl. Supernatant (140 μl) was then used for viral RNA extraction using QIAamp Viral RNA Mini Kits (Qiagen) according to the manufacturer’s instructions.

cDNA samples were prepared from 10 μl of the extracted RNA samples using a High Capacity cDNA reverse transcription kit (Applied Biosystems), and 5 μl of the cDNA product was used as a template for RT-qPCR using either Power SYBR-Green (Applied Biosystems) or Taqman Fast (Applied Biosystems) detection chemistries. The primer and probe sequences used in RT-qPCR reactions are specific to the ZIKV NS5 coding region conserved between the African and Asian ZIKV lineages, and are designed to account for sequence variability between sequenced ZIKV strains [25] (Table 1). For generating a standard curve, DNA corresponding to a fragment of the ZIKV NS5 gene was synthesized *in vitro* (IDT gBlock) using sequence from the Asian lineage Z1106033 strain (Suriname, accession number KU312312 [26]). Standard curves were generated for each separate RT-qPCR experiment to quantify ZIKV levels. RT-qPCR was performed on a ViiA 7 Real-Time PCR system (Applied Biosystems) at the University of Texas at Austin Genomic Sequencing and Analysis Facility (GSAF). Sanger dye-terminator sequencing was performed at the GSAF to confirm the RT-qPCR product.

**Table 1:** Sequence of primers and probe.

		Sequence	
Forward	5’	AARTACACATACCARAACAAAGTGGT	3’
Probe	5’	FAM-CTYAGACCAGCTGAAR-MGB	3’
Reverse	5’	TCCRCTCCCYCTYTGGTCTTG	3’

## Results

**Fig 1:**
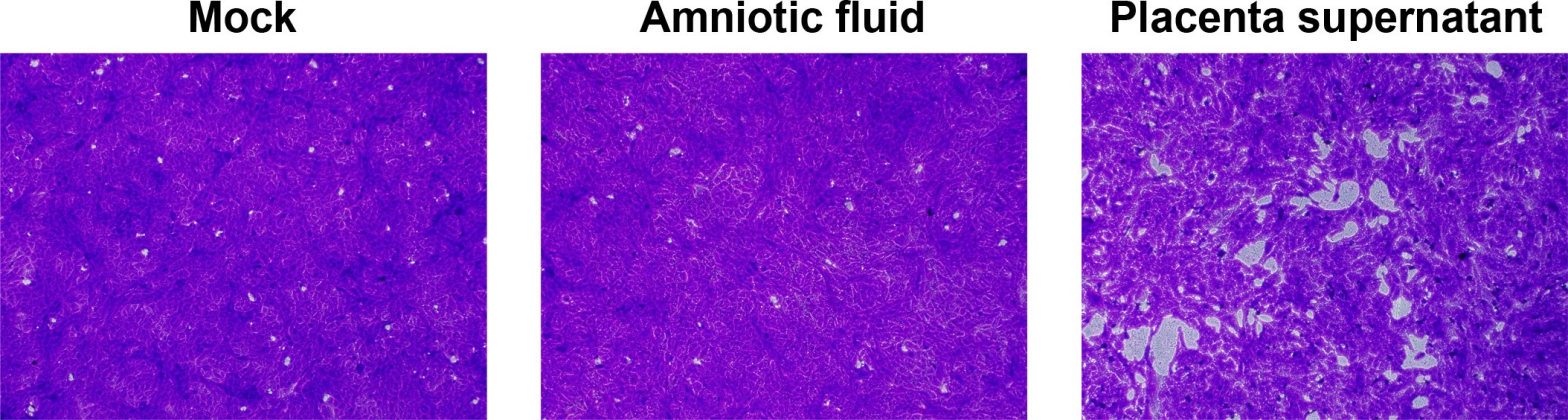
ZIKV plaque formation Vero cells. Amniotic fluid and placental supernatant were collected by C-section from animal #5574 at 26 days post infection (dpi) following detection of intrauterine fetal demise. Amniotic fluid added to Vero cell monolayers produced no observable lesions, whereas placental supernatant produced plaques. Monolayers were stained with crystal violet, and images were taken with a 10x objective.

Squirrel monkeys are an established nonhuman primate model of ZIKV pathogenesis [19], and we used samples collected from two experimentally-infected pregnant females (designated animal #5165 and animal #5574) and two control animals to test the feasibility of ZIKV detection in feces. At 26 days post infection (dpi), animal #5574 experienced intrauterine fetal demise, based on failure to detect heartbeat activity or fetal movement by high-resolution ultrasound. A C-section was performed to recover amniotic fluid and placental tissue. Culture of an extract from placental tissue on Vero cells resulted in plaque formation at 72 hours, but amniotic fluid did not (Fig 1). The plaque assays provide independent confirmation that the inoculation with ZIKV was successful.

**Fig 2:**
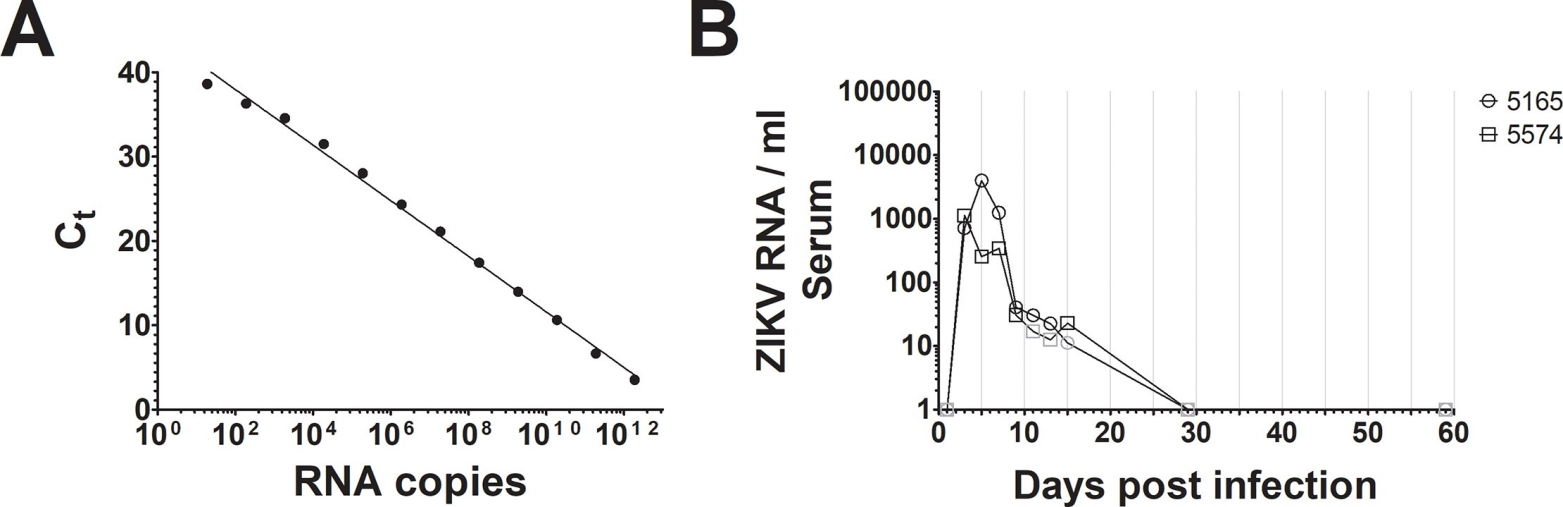
(A) A representative standard curve for ZIKV detection by RT-qPCR. A portion of the ZIKV NS5 gene synthesized *in vitro* was used to generate a RT-qPCR standard curve using SYBR-Green. A non-linear regression was used to determine correlation (R^2^ = 0.99). (B) RNA was extracted from the serum sampled from two experimentally infected non-human primates over time, and ZIKV was quantified using RT-qPCR. ZIKV was detected between 3–15 dpi. Gray symbols indicate ZIKV at levels outside the range of the standard curve. No ZIKV was detected at 59 dpi.

We sought to develop a high-throughput method to detect ZIKV infection in captive primates that would be applicable to ZIKV detection in wild primates. To test for ZIKV in the inoculated squirrel monkeys, we collected serum, saliva, urine, and fresh feces during variable time points over a period of 59 days. RT-qPCR was selected for testing as a scalable, cost-effective, and validated ZIKV detection method. To determine if RT-qPCR detected ZIKV viremia in our subjects, we extracted viral RNA to perform RT-qPCR with a previously documented primer set [25]. As a positive control, a fragment of the ZIKV NS5 gene was synthesized *in vitro*. A known concentration of this DNA product was serially diluted to produce a standard curve for ZIKV quantification, demonstrating linear detection as low as approximately 20 NS5 copies per reaction (Fig 2A). Using RT-qPCR and the SYBR-Green detection chemistry, ZIKV was detected in serum samples of both of the experimentally infected squirrel monkeys 3 days post infection, confirming ZIKV viremia (Fig 2B). ZIKV continued to be detectable within the range of our standard curve at 13 dpi in animal #5165 and 15 dpi in animal #5574. Between days 11–13 for animal #5574 and on day 15 for animal #5165, we observed ZIKV RNA levels outside the range of our standard curve; quantities were extrapolated from our standard curve and we consider these likely positives (indicated in gray). Serum RNA levels were higher in animal #5165 compared to animal #5574 for the majority of the infection, indicating previously described variability in experimental ZIKV infection of squirrel monkeys [19].

**Fig 3:**
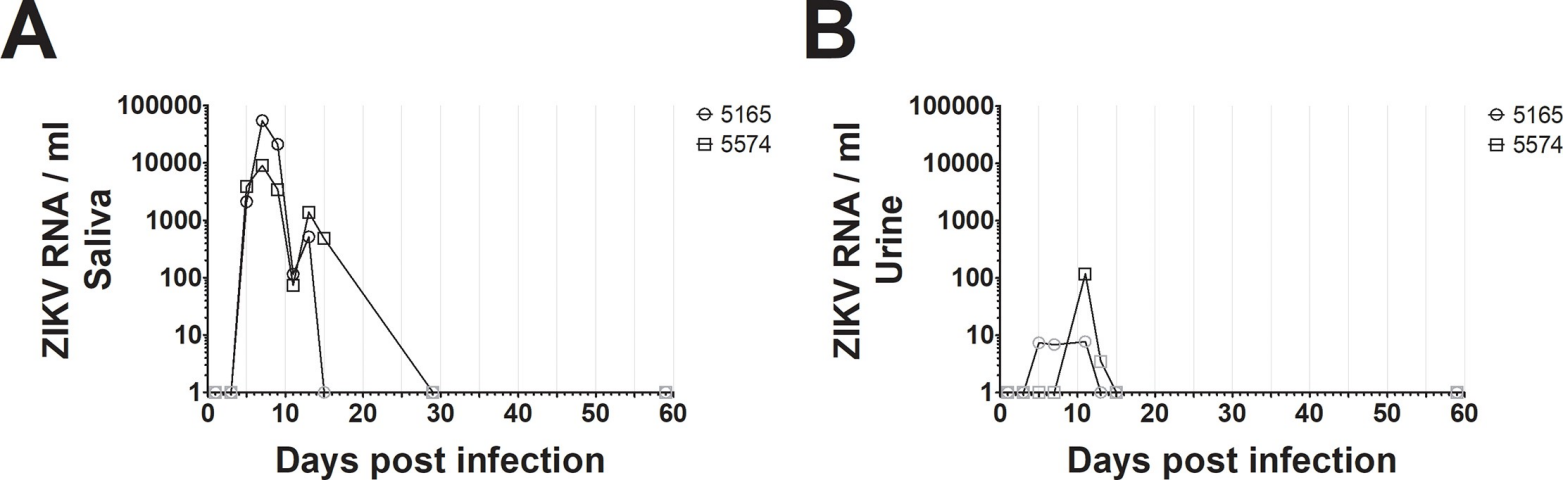
ZIKV was quantified in saliva (A) and urine (B) samples from experimentally infected non-human primates using SYBR-green RT-qPCR. ZIKV was detected between days 5–15 in saliva, and on day 11 in urine. These data are representative of two experiments from independent RNA extractions. Gray symbols indicate ZIKV at levels outside the range of the standard curve. No ZIKV was detected at 59 dpi in either saliva or urine.

Because ZIKV viremia is detectable at various levels in different tissues over the course of infection, we also examined saliva and urine samples from our two subjects for the presence of ZIKV RNA. We detected ZIKV by RT-qPCR in saliva from both animals as early as 5 dpi, continuing until days 13–15. ZIKV levels were lower for the majority of the course of infection in squirrel monkey 5574 compared to 5165 (Fig 3A).

The presence of ZIKV in urine has been shown to offer longer and more sensitive periods of detection in human patient samples [27]. ZIKV was detectable in urine samples by RT-qPCR within the range of our standard curve starting only at day 11 in animal #5574, however we observed ZIKV levels outside the range of our standard curve between days 5–13 (Fig 3B). Viral RNA levels in urine were also substantially lower compared to those seen in serum and saliva samples. Nonetheless, these data indicated that our assay detected ZIKV in multiple sample types as observed in other primates [18,19].

**Fig 4:**
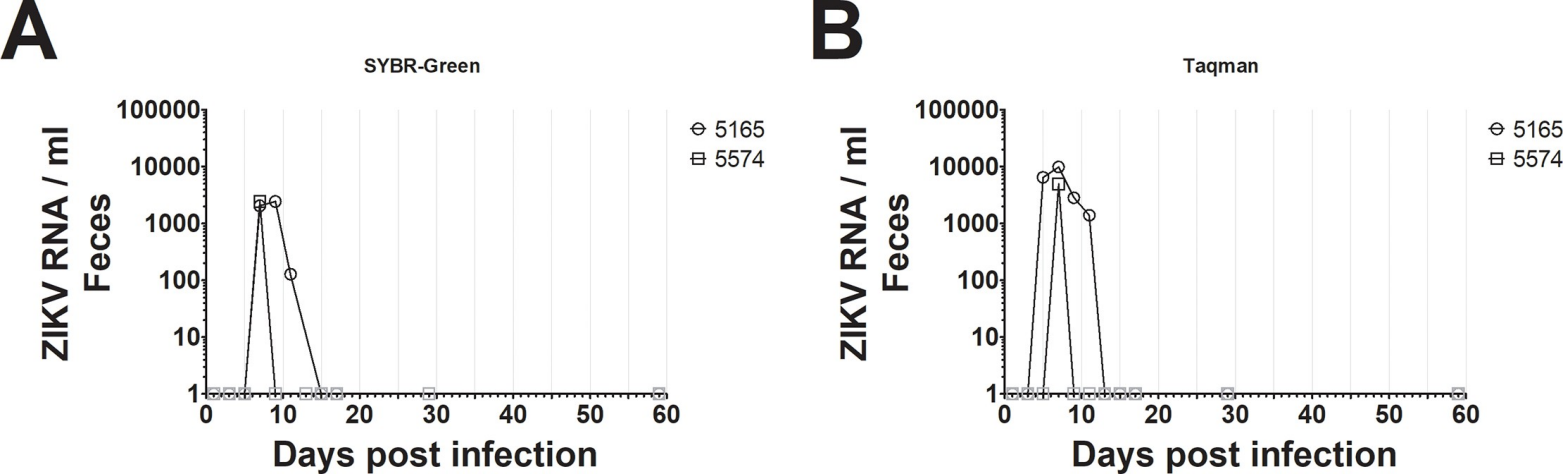
ZIKV was quantified in fecal samples from experimentally infected non-human primates using RT-qPCR. (A) SYBR-green was used to quantify ZIKV in fecal samples. ZIKV was detected between 7–11 dpi. (B) Taqman was used to quantify ZIKV in fecal samples. ZIKV was detected between 5–11 dpi. Gray symbols indicate ZIKV at levels outside the range of the standard curve. No ZIKV was detected at 59 dpi using either SYBR-Green or Taqman, and no ZIKV was detected in feces of uninfected individuals.

Finally, we used RT-qPCR to determine the feasibility of detecting ZIKV in fecal samples. We tested for ZIKV in RNA extracted from feces by RT-qPCR from the two inoculated subjects using the SYBR-Green methodology described above, as well as from fecal samples of two uninfected squirrel monkeys as a negative control. Because viral recovery from feces is contingent on several dynamic factors including diet, time between feeding and defecation, time between defecation and collection, and potential environmental contamination, we consider this method semi-quantitative. ZIKV was detected between days 7–11 post-infection in the fecal samples from animal #5165 (Fig 4A). As expected, ZIKV was detected in animal #5574 only on day 7, consistent with the lower infection levels we observed for this subject in serum, urine, and saliva. The PCR product melting temperatures were consistent between the fecal samples and other sample types. ZIKV was not detected for the second pair of uninfected squirrel monkeys. Because SYBR-Green nonspecifically detects DNA amplification from cDNA, we confirmed that ZIKV RNA was present in the sample by sequencing the RT-qPCR product for animal #5165 (day 7). The non-primer sequence from both complementary strands of the PCR product mapped to the predicted NS5 gene of the ZIKV genome.

To increase the sensitivity of ZIKV detection in fecal samples, we attempted using the same primer set as above and a ZIKV NS5 Taqman probe in conjunction with RT-qPCR (Table 1). Although less cost efficient, Taqman probes are typically more sensitive than SYBR-Green, and are less prone to false positive results. ZIKV was detected beginning on day 5 in animal #5165 using Taqman probes, two days earlier than using SYBR-Green (Fig 4B). Similar to the SYBR-Green results, ZIKV was only detected on day 7 in animal #5574 using the Taqman probe. Additionally, we observed higher inferred concentrations of ZIKV in both subjects using the Taqman probe compared to SYBR-Green, indicating that the sensitivity of our detection was enhanced.

## Discussion

The degree of overlap between nonhuman primate habitats and human populations in the Americas, particularly as a result of habitat fragmentation and other anthropogenic changes to wild primate habitats, makes zoonotic transmission of ZIKV a significant public health risk. For closely related flaviviruses, such as YFV and DENV, transmission from nonhuman primate hosts to humans by mosquitoes is a major concern for human health [11,13,28]. This pattern of flavivirus transmission suggests that Neotropical primates could also serve as important sylvatic reservoirs of ZIKV, which can be transmitted between species through mosquito vectors that range across most of Latin America, the southern United States, and even extending to the Great Lakes [29]. Thus, zoonotic transmission of ZIKV from potential nonhuman primate reservoirs is possible in most parts of the Americas where humans and nonhuman primates overlap.

In Brazil, antibodies against ZIKV have been detected in samples from black-striped capuchin (*Sapajus libidinosus*) [20], blond capuchin (*Sapajus flavius*) [20,21], red-handed howler monkeys (*Alouatta belzebuth*) [21], and black-tufted marmosets (*Callithrix penicillata*) [21], suggesting the possibility of anthroponotic transmission, but these results are inconclusive because of the high cross-reactivity among flavivirus antibodies. In a non-refereed report, Favoretto et al. [30] used PCR to detect ZIKV in blood and saliva samples from wild common marmosets (*Callithrix jacchus*) and black-striped capuchin monkeys (*Sapajus libidinosus*) in Brazil and found that the strain of ZIKV found in these primates was identical to the strain circulating in human populations in the Americas. Improved ZIKV surveillance requires verification of the strain carried by Neotropical primates, identification of the geographic range of the disease, and development of specific and efficient detection methods.

We demonstrate here that ZIKV can be detected in feces of squirrel monkeys following experimental infection using a simple, cost-effective, RT-qPCR based assay. One other study has detected ZIKV in nonhuman primate feces [18]; however, that study examined a different strain of ZIKV (the original 1947 Uganda African lineage) in a different neotropical primate species (the common marmoset, *Callithrix jacchus*), and was only conducted on males. In that study, the marmosets also showed variation in detection patterns between subjects and sample types, but generally followed a similar pattern to what we observed here. One notable difference was that ZIKV was only detected in the blood of the marmosets as late as day 7, whereas in our study ZIKV was detected in the blood of squirrel monkeys until day 15; however, the longer lasting viremia we noted could also be due to gender differences or pregnancy rather than species differences [31]. The period of ZIKV detection in the feces of marmosets was similar to that observed here, between days 5–13 in marmosets [18] and days 5–11 in squirrel monkeys. Individual variation in viremia is also likely, as we observed differences in the number of days ZIKV could be detected in feces between our two individuals, with one animal having a positive fecal sample only on day 7 (Fig 4). Importantly, our results confirm a previous study of ZIKV fecal detection [18], extend these findings to the Mexican strain of the virus and to an additional species/sex combination of primate, and add important methodological details for others seeking to monitor ZIKV in Neotropical primates. We also demonstrate that the highly conserved ZIKV NS5 gene can be used as a target for PCR-based detection in feces, rather than envelope sequences [18]. These findings indicate that fecal detection of ZIKV is plausible in multiple species of Neotropical primates, and that fecal detection of ZIKV in Neotropical primate samples is a viable method for noninvasively monitoring the disease in the wild, with the potential for an extended detection time.

The use of RT-qPCR for ZIKV detection is a cost effective and high throughput method for monitoring ZIKV infection in wild nonhuman primates. Although shorter than the ZIKV detection period in serum for squirrel monkeys, fecal ZIKV detection is possible for a portion of the time that ZIKV is detected in blood and is a non-invasive method for assessing infection in wild populations, although the cellular origin of the virus in feces is unclear. Our methodology, as described here, is best considered semi-quantitative, due to the fact that fecal content is immensely variable, especially in wild primates, and because ZIKV detection in feces was not normalized to the mass of source material. Nevertheless, this approach eases the burden of field collection and increases throughput, but could easily be modified to yield more quantitative results.

Further studies will need to be conducted to determine the impact of field conditions on the preservation of virus in fecal materials; however, current practices of primatologists to collect fresh feces and store them frozen or in a nucleic acid preservation buffer will be sufficient to preserve the virus in these samples. It is common practice for scientists studying nonhuman primates in the wild to have teams that follow the animals on a daily basis and collect samples from known individuals. These methods allow collection of fecal samples that are regularly preserved and processed for laboratory analyses for hormonal, genetic, and disease studies [32–34], and the methods can be modified to monitor ZIKV in study populations. Importantly, researchers will also now have a noninvasive way to check for ZIKV, particularly if reproductive issues are observed or if an infection is suspected. During these times, researchers can increase sample collection frequency to increase the likelihood of detecting the virus. We recommend using Taqman assays rather than SYBR-Green to improve detection sensitivity and specificity. Many long-term Neotropical primate projects exist throughout Latin America, including those that have been studying the same groups of monkeys for over a decade; the methods described here may provide an important tool for these research teams to examine the impact of an emerging disease within their study areas.

Considerable variation in flavivirus susceptibility and the associated symptoms of flavivirus infections is demonstrable in nonhuman primates. For example, compared to infection of Old World primates, YFV presents with more severe symptoms in Neotropical monkeys [35] and has caused the death of hundreds of wild howler monkeys (*Alouatta spp*.) in Argentina [16] and Brazil [36,37]. Although many species within the Neotropical genera *Alouatta*, *Ateles*, *Saimiri*, and *Aotus* commonly die from YFV, species of *Cebus* and *Lagothrix* do not [38–40]. This variation in susceptibility to or severity of flaviviral disease in Neotropical primates could mean that specific neotropical primate species may be more impacted by the current ZIKV epidemic (e.g., fetal pathology, as observed by Vanchiere et al. [19]). Such results have implications for both human health and nonhuman primate conservation. Further studies are necessary to determine the factors that promote variability in ZIKV fecal shedding in infected nonhuman primates. This case study was limited by small sample size (n = 2) and by the use of pregnant females that may have had prolonged viremia and increased shedding of the virus. Although our findings are important for potentially detecting the virus in pregnant females in the wild, additional studies should be conducted to compare these results to non-pregnant individuals. Wild Neotropical primates are likely sylvatic reservoirs of ZIKV in the Americas, making it critical to develop methods such as these to effectively monitor their populations for infection.
